# Development and Validation of a 34-Gene Inherited Cancer Predisposition Panel Using Next-Generation Sequencing

**DOI:** 10.1155/2020/3289023

**Published:** 2020-01-22

**Authors:** Sun Hee Rosenthal, Weimin Sun, Ke Zhang, Yan Liu, Quoclinh Nguyen, Anna Gerasimova, Camille Nery, Linda Cheng, Carolyn Castonguay, Elaine Hiller, James Li, Christopher Elzinga, David Wolfson, Alla Smolgovsky, Rebecca Chen, Arlene Buller-Burckle, Joseph Catanese, Andrew Grupe, Felicitas Lacbawan, Renius Owen

**Affiliations:** ^1^Department of Genetics, Quest Diagnostics Nichols Institute, San Juan Capistrano, CA 92675, USA; ^2^Athena/Quest Diagnostics, Marlborough, MA 01752, USA

## Abstract

The use of genetic testing to identify individuals with hereditary cancer syndromes has been widely adopted by clinicians for management of inherited cancer risk. The objective of this study was to develop and validate a 34-gene inherited cancer predisposition panel using targeted capture-based next-generation sequencing (NGS). The panel incorporates genes underlying well-characterized cancer syndromes, such as *BRCA1* and *BRCA2* (*BRCA1/2*), along with more recently discovered genes associated with increased cancer risk. We performed a validation study on 133 unique specimens, including 33 with known variant status; known variants included single nucleotide variants (SNVs) and small insertions and deletions (Indels), as well as copy-number variants (CNVs). The analytical validation study achieved 100% sensitivity and specificity for SNVs and small Indels, with 100% sensitivity and 98.0% specificity for CNVs using in-house developed CNV flagging algorithm. We employed a microarray comparative genomic hybridization (aCGH) method for all specimens that the algorithm flags as CNV-positive for confirmation. In combination with aCGH confirmation, CNV detection specificity improved to 100%. We additionally report results of the first 500 consecutive specimens submitted for clinical testing with the 34-gene panel, identifying 53 deleterious variants in 13 genes in 49 individuals. Half of the detected pathogenic/likely pathogenic variants were found in *BRCA1* (23%), *BRCA2* (23%), or the Lynch syndrome-associated genes *PMS2* (4%) and *MLH1* (2%). The other half were detected in 9 other genes:* MUTYH *(17%)*, CHEK2 *(15%),* ATM *(4%),* PALB2 *(4%),* BARD1 *(2%),* CDH1 *(2%),* CDKN2A *(2%),* RAD51C *(2%), and * RET *(2%). Our validation studies and initial clinical data demonstrate that a 34-gene inherited cancer predisposition panel can provide clinically significant information for cancer risk assessment.

## 1. Introduction

It is important to identify individuals with hereditary cancer efficiently and in a cost-effective manner, as they account for 5–10% of all cancers [1]. Over the last few years, rapid advances in next-generation sequencing (NGS) technologies have allowed simultaneous interrogation of multiple genes. NGS technologies perform at higher throughput than Sanger sequencing, since they work in a massively parallel manner [2]. As a result, multigene panel tests utilizing NGS can be a cost-effective and efficient way to detect clinically actionable mutations in appropriately selected patients [3]. Their use may increase detection of pathogenic mutations compared to single-gene testing [4].

Identifying hereditary cancer susceptibility in an individual with a personal and/or family history can be complex. Pathogenic/likely pathogenic variants in multiple genes can be associated with cancer in a specific organ. For example, ovarian cancer is associated with pathogenic/likely pathogenic variants in multiple genes such as *BRCA1*, *BRCA2*, *MLH1*, *MSH2*, *RAD51C*, and *RAD51D* [5–11]. On the other hand, pathogenic/likely pathogenic variants in a single gene can increase the risk of more than 1 type of cancer. Women with Lynch syndrome due to *MLH1 *pathogenic variants have an increased risk of specific cancers, including cancer of the colon or rectum (52–82%), uterus (25–60%), ovary (4–24%), stomach, urinary tract, pancreas, small bowel, hepatobiliary tract, and brain [7–9, 12–19].

Several professional societies have published guidelines that support and define genetic testing for hereditary cancer syndromes. These societies acknowledge that multigene panel testing may benefit individuals when their histories are consistent with multiple possible hereditary cancer syndromes or when a syndrome can be caused by multiple genes [20, 21]. These panels are also informative for families with a limited structure, when family history information is limited, and when the history of cancer is strong but targeted testing has been negative [18, 22–27]. Guidelines, however, do not currently provide guidance on when multigene panel testing may provide actionable additional information for individuals whose histories do not meet these criteria.

Here, we describe the development and validation of a 34-gene inherited cancer predisposition panel using NGS for single-nucleotide variants (SNVs), insertions and deletions (Indels), and exon-level copy number variants (CNVs). We also studied variant detection yield of the panel by summarizing deidentified results from 500 consecutive patient specimens submitted for clinical testing with the 34-gene panel.

## 2. Materials and Methods

### 2.1. Gene Selection

The 34-genes selected for this panel are associated with well characterized cancer syndromes, along with more recently discovered genes associated with increased cancer risk (Table 1). The genes selected increase the lifetime risk of cancer of the breast, ovary, colon, rectum, endometrium, pancreas, prostate, neuroendocrine system, and/or other cancer types. Genes were selected that conferred at least a twofold increased risk of cancer or a 5% lifetime risk to develop cancer. Additionally, at the time of test development, genetic testing and/or medical management guidelines existed for many of the individual genes on the panel [18, 24–26, 28]. Common polymorphism contributions to a polygenic risk score were excluded from this panel design.

### 2.2. DNA Specimens

For validation of the 34-gene hereditary cancer predisposition panel, 131 de-identified residual whole blood patient specimens submitted for routine clinical testing were used. In addition, a specimen positive for a 40-base deletion in *BRCA1* (GM14094) and the well-characterized NA12878 DNA specimen were tested (Coriell Mutant Cell Repository, Camden, NJ).

To assess variant detection yield of the 34-gene panel in practice, we analyzed findings from the first 500 clinical specimens submitted for testing with this assay. For each patient, informed consent for genetic analysis was obtained. Patient results were de-identified before analysis.

### 2.3. Next Generation Sequencing

Genomic DNA from whole blood or cultured cells was isolated using the Roche Magnapure system (Roche Molecular Systems, Indianapolis, IN). Isolated genomic DNA was mechanically sheared to an average size of 250 bases using a Covaris instrument LE220 (Covaris Inc., Woburn, MA). The fragmented DNA was enzymatically repaired and end-modified with adenosine (NEBNext Ultra DNA Library prep kit, NEB, Ipswich, MA) to make it receptive to T/A ligation with barcoded adapters (Integrated DNA Technologies, Coral, IL). The ligated products were size-selected (AMPure Beads, Agencourt, Beverley, MA) and amplified (GeneRead DNA I Amp Kit, Qiagen, Mississauga, ON) and then the regions of interest were captured using biotinylated RNA baits (SureSelect, Agilent, Mississauga, ON). The baits were designed to capture all coding exons and exon/intron boundaries of the 34 hereditary cancer related genes (Table 1). In addition, any noncoding regions of these genes containing currently known pathogenic variants, as well as the promoter regions of *APC, MLH1, MSH2, *and* PTEN* were included. The DNA/RNA hybrids were enriched with streptavidin attached magnet beads (Dynabeads MyONe Streptavidin T1, Thermo Fisher Scientific, Markham, ON) and subjected to washing under increasing stringency in order to remove non-targeted DNA sequences. A second amplification was performed (Herculase ® II Fusion DNA Polymerase, Agilent, Mississauga, ON), followed by bead purification (AMPure Beads, Agencourt, Beverley, MA) to remove all unused primers and nucleotides.

To achieve assay specificity by avoiding interference from pseudogenes, exons 11–15 from *PMS2* and *CHEK2* were amplified from genomic DNA by long-range PCR (LR–PCR, Takara LA Taq DNA polymerase, Takara Bio, Mountain View, CA). For *PMS2 *amplification, primers PMS2–LR3F (forward; within exon 10, sequence from Clendenning et al [29]) and PMS2–LR3Rm (reverse; ~1.6 kb downstream of exon 15) were used. For *CHEK2* amplification, primers CHEK2–Fm3 (forward; ~0.6 kb downstream of exon 15) and CHEK2-R (reverse; within intron 10) were used. All primer sequences are listed in Supplemental [Supplementary-material supplementary-material-1]. LR–PCR products were subjected to mechanical shearing using a Covaris E220 instrument, enzymatic end repair, and 3′ adenylation, followed by ligation to barcoded adaptors and a second PCR to enrich ligated fragments as described above. Final products from the LR–PCR library and the captured gDNA library were combined and sequenced on an Illumina NextSeq instrument, 2 × 150 cycles (NextSeq500 mid output v2 kit, Illumina, San Diego, CA).

### 2.4. Bioinformatics Processing

Following the sequencing reaction, sequence alignment and allele assignment were performed. BCL files from NextSeq500 were converted to FASTQ files. The raw sequence reads in FASTQ files were then aligned to the Genome Reference Consortium human genome build 37 (GRCh37), or custom reference genome, using the Burrows–Wheeler Aligner (BWA). The custom reference genome differs from GRCh37 in that highly homologous pseudogene sequences within chr22:16,983,750–16,990,200 and chr7:6,776,750–6,791,250 were replaced with nucleotide T for accurate alignment of LR–PCR library to the *CHEK2* and *PMS2* gene regions. Mapped reads were filtered by Phred Quality score of read mapping over 30 (>99.9% accuracy), before downstream analysis. Reads were then sorted and indexed using SAMtools, followed by removal of read duplications using Picard Tools. Local realignment and base-quality score recalibration were performed using the Genome Analysis Toolkit (GATK). Average and minimum depth of coverage for every region of interest (ROI) were computed, and variant calling was performed using GATK Unified Genotyper and Haplotyper. A single variant call file (vcf) was created by merging variant call files from both variant callers. Coverage and variant-depth reports were created and loaded to the sequencing database (seqDB). Alamut Batch was used to obtain high-level annotation for detected variants. For *EPCAM*, only copy number variant analysis was performed. Each targeted base within the ROI (115,483 bp) was required to have at least 20x unique reads.

### 2.5. Copy Number Variant Analysis

CNV-positive specimens were identified using a CNV flagging algorithm developed in-house based on the well-established NGS read-depth (RD) approach [30, 31]. To calculate the read-depth, we partitioned large targets into nonoverlapping bins of approximately 200 bp and used the average read-depth of each bin. To enhance large CNV signals (e.g. *BRCA1 *exon 9–12 del, whole gene del/dup), we generated additional target groups by joining multiple bins within the target range. Each target group was considered independently. For CNV target flagging, a normalized target representation value *X* was calculated using the following steps: (a) target read-depth was divided by mean read-depth of all targets to give relative target read-depth, *RDr*; (b) principle component analysis (PCA) was performed on the *RDr*, generating the *RDrp; *(c) *RDrp* was further normalized by dividing the target *RDrp* by the median *RDrp* of all targets, yielding *X*. The *Z*score of each target was calculated from the following formula: *Z* = (*X* − *μ*)/*σ*, where *X* is normalized *RDrp*, *μ* is the median *X* across specimens in a batch typically composed of 92 specimens, and *σ* is the median absolute deviation of *X* from a training set of about 1,300 specimens. To augment the *Z* score, the *Z.adj* score was used; the *Z.adj* score was calculated by dividing the *Z* score by the standard deviation of *Z* score across targets, excluding the given target. This *Z.adj* extracts putative CNV signal from noisy read-depth to discern patterns of read depth biases. Finally, a CNV target was flagged if the target *Z* and/or *Z.adj* for a given target group were over the cut-off value created from a training set of approximately 8,000 specimens. *PMS2* and *CHEK2* exons 11–15 were excluded from copy number analysis.

### 2.6. Array Comparative Genomic Hybridization

An aCGH method was used to complement the NGS CNV flagging algorithm. All specimens flagged as having CNVs by NGS were reflexed to aCGH for CNV confirmation. Custom array probes for the 34-genes in the panel were designed using Agilent SureDesign custom design tool. Approximately 57,000 probes were designed, giving on average 26 probes per exon with less density for introns and promoters. Patient specimens and gender-matched reference specimens were labeled with Cy5 and Cy3, respectively, using Agilent SureTag Complete DNA Labeling Kit followed by column purification and volume reduction. The labeled DNA was then combined and hybridized onto a custom microarray slide (Agilent, Mississauga, ON). After hybridization, the slides were washed to remove non-specific binding and then immediately scanned on the Agilent SureScan or C scanner; data were extracted using Agilent CytoGenomics Software. The results were manually reviewed by licensed personnel and a licensed director for report generation.

### 2.7. Variant Assessment

All variants detected by the NGS panel were manually reviewed by licensed personnel and classified by a team of variant scientists as described previously [32], according to the ACMG guidelines [33]. Classifications were scored as benign, likely benign, unknown significance (VUS), likely pathogenic, or pathogenic. This result was then reviewed by a licensed director for reporting. Variants identified through clinical testing at our laboratory are routinely submitted to ClinVar [34]. The variant of uncertain significance rate for the first 500 clinical samples was calculated based on the percentage of patients tested with no pathogenic or likely pathogenic variants identified and 1 or more VUSs identified. For this study, variants are considered clinically actionable when classified as either pathogenic or likely pathogenic.

## 3. Results

### 3.1. Assay Performance

The 133 unique specimens included in the assay validation study were sequenced for a total of 726 trials in 8 consecutive sequencing runs. We aimed minimum unique sequencing coverage of 50x for 100% of our targeted region of interest. For routine analysis, coverage of 20x is required for all bases in the targeted region. In the validation runs, 99.8% of the targeted regions were covered 100x, with an average median coverage depth of 382x (range: 89–946). All target regions within coding exons were covered over 100x. Among the 726 tested specimens, 99.9% specimens passed coverage requirement of 20x with on average 380x median coverage depth (range: 112–689).

### 3.2. Technical Validation for SNVs and Indels

Sequencing variant detection accuracy was assessed using 15 specimens with collectively 56 known variants (28 unique) from bidirectional Sanger sequencing in at least one of the following genes: *APC, MLH1, MSH2, MSH6, PMS2*, and *RET*. The 28 unique variants included 23 SNVs and 5 deletions. In addition, a *BRCA1* c.1175_1214del40 positive specimen GM14094 was tested. All expected 57 variants were accurately detected including the 40 bp deletion in *BRCA1* (Table 2). Subsequently, a well-characterized HapMap reference specimen (NA12878) was investigated for the regions with a highly confident published consensus sequence overlapping with our ROI. Out of a total of 91,940 bp tested, all 45 known benign variants were detected, with no false-positive calls (data not shown). All specimens were replicated 9 times, except the *BRCA1* c.1175_1214del40 positive control, which repeated 5 times. Collectively, the assay yielded 100% accuracy for detection of known sequencing variants.

To test intra- and inter-assay reproducibility, a set of 30 specimens was analyzed, each in triplicate within a run and repeated three times, for a total of 9 replicates per specimen. The specimens selected for reproducibility studies comprised 15 specimens with known variants used in the accuracy study described above, NA12878, and 14 de-identified specimens with unknown genotype. In all, within reportable ROI, NGS detected 1,733 variants (228 unique) from the 30 specimens. These included 1,708 SNVs (216 unique) and 25 indels (12 unique). Of these, 1,418 variants (179 unique) were in coding exons and 315 variants (49 unique) were in nonexonic regions. It is important to note that there were 4 discordant positions in 115,487 sequenced bases among the 9 trials per specimen. Those positions were residing within homopolymeric regions which are known to cause sequencing artefacts. As all discordant or inconsistent variant calls occurred in nonexonic regions with no known clinical significance, variants observed in those 4 positions were excluded from the reportable range. Within reportable ROI, there were 100% intra- and inter-assay variant call concordances from all 9 trials per specimen.

### 3.3. Technical Validation for CNVs

To validate the in-house developed CNV flagging algorithm, a set of 103 anonymized DNA specimens with known CNV status were tested for a total of 457 trials. These included 18 CNV positive specimens by either MLPA or aCGH (Table 3).

Intra-assay precision studies included 2 CNV positive specimens (S8 and S10, Table 3), replicated 5 and 3 times, respectively, and a CNV negative specimen replicated 5 times. Each of the replicates gave concordant CNV call resulting in 100% intra-assay precision. Inter-assay precision studies included 94 de-identified specimens replicated 2 (*n*=1) or 3 times (*n*=93) for a total of 281 trials. Of these, 9 were known positives (S1–S9, Table 3). Including the 9 known CNV positive specimens, 90 specimens showed concordant CNV calls among replicates, yielding 95.7% (90/94) inter-assay precision. 4 specimens resulted in discordant calls in at least one of the replicates. All specimens flagged as having CNVs by NGS were reflexed to aCGH for CNV confirmation. All discordant specimens were CNV negative by aCGH. In combination with aCGH confirmation, the inter-assay precision was improved to 100%.

All 18 positive specimens were correctly called in a total of 42 trials, yielding 100% CNV detection sensitivity. Three of the 18 positives had a single-exon duplication or deletion, which is known to be challenging to detect by a targeted NGS assay. CNV detection specificity was evaluated from 457 trials of 103 unique specimens. There were 9 false-positive calls from 6 unique specimens including the 4 specimens described in inter-assay precision study, resulting in 98.0% (448/457) assay specificity. In combination with aCGH confirmation, the CNV detection specificity was 100%.

### 3.4. The First 500 Clinical Specimens

The validated 34-gene panel for variants associated with inherited cancer predisposition was applied to the molecular diagnosis of 500 consecutive, unique de-identified patient specimens. Based on the clinical information submitted, indications for testing included a personal and/or familial history of cancer—most frequently breast, ovarian, or colon. The assay detected a total of 51 pathogenic and 2 likely pathogenic variants in specimens from 49 (9.8%) of the 500 patients. For the first 500 specimens, the panel yielded at VUS rate of 36%. The classification of each variant reflects the classification at the time of reporting. Among the 53 pathogenic/likely pathogenic variants detected, 48 were within exons (26 SNVs and 22 indels), 3 were in splice junctions (3 SNVs), and 2 were CNV variants (a *BRCA1* exon 13 duplication, and a *PMS2* deletion of exon 7 and 8). The relative distribution of the 53 pathogenic/likely pathogenic variants detected in this study is provided in Table 4. *BRCA1/2* were the most frequently mutated genes, with 21 patients harboring 24 pathogenic variants. In addition, 3 patients had pathogenic variants in the Lynch syndrome-associated genes *PMS2* (*n* = 2) and *MLH1* (*n* = 1). The remaining 26 patients had pathogenic/likely pathogenic variants in 9 other genes: monoallelic *MUTYH* variants in 9 patients; *CHEK2 *variants in 8; *ATM*, *CDH1*, and *PALB2* variants in 2 patients each, and *BARD1*, *CDKN2A*, *RAD51C*, and *RET* variants in 1 patient each. Notably, 4 patients had 2 pathogenic variants each: 3 had pathogenic variants in *BRCA1/2*, and 1 had pathogenic variants in *BRCA2 *and *MUTYH*.

## 4. Discussion

Various targeted NGS-based multigene inherited cancer panels have been developed by clinical diagnostic laboratories [14, 18–22]. As each laboratory-developed test uses different laboratory procedures and bioinformatics pipelines, rigorous laboratory validation is critical to ensure accurate and reliable results from NGS assays for use in clinical practice [35]. In this study, we demonstrated that the 34-gene cancer predisposition panel achieved 100% analytical sensitivity and specificity for SNVs and small Indels. A well-known challenge for NGS is accurate variant calling in low-complexity and homopolymeric regions. From our inter- and intra-assay precision studies, we identified 4 different positions within homopolymeric context causing discordant variant calls and excluded from reportable range. While one of those positions resulted in 9 false variant calls (31% median variant frequency) out of 270 tested cases, the remaining three resulted in only 1 or 2 false calls (23% median variant frequency). This result suggests the importance of using multiple specimens for precision studies to define reportable range for an NGS assay. A pathogenic splice-site variant, *MSH2* c.942 + 3A > T, is known to be difficult to detect due to the presence of an adjacent 27 bp polyA sequence. By using a customized GATK Unified Genotyper setting to force calls at each site, we detected 20 cases of *MSH2* c.942 + 3A > T from 51,129 clinical results of the 34-gene cancer predisposition panel. In comparison, 5 of these cases were not detected with the default Unified Genotyper setting. Therefore, the characteristics of sequence context should be considered for developing an appropriate analysis pipeline.

The presence of pseudogenes can also interfere with variant calling. Accurate molecular testing of the *PMS2* 3ʹ region is complicated by the pseudogene *PMS2CL, *a partial duplication of *PMS2* (exon 9, and 11–15), located ~0.7 Mb proximal to *PMS2* as a result of an inverted duplication of a 100 kb repeat element. To avoid interference from the pseudogene, a *PMS2* exon 11–15 sequencing library was prepared from long-range PCR fragments amplified using primers specific to either *PMS2* or the *PMS2/PMS2CL* hybrid allele in the event of a gene conversion [36]. Nonetheless, during development we found a false-negative NGS call for a Sanger sequencing-confirmed variant, c.2466 T > C (rs10000, benign) in *PMS2* exon 15. The false-negative call was caused by an adjacent c.∗92dupA variant (VUS, pseudogene specific sequence) resulting in preferential sequence alignment to *PMS2CL *reference sequence. It has been reported that sequence exchange between *PMS2* and *PMS2CL* has led to considerable sequence homogenization, and as a result, pseudogene specific variants according to the reference sequence are also found in real gene, and vice versa [36]. The correct call for c.2466 T > C was made upon using the custom reference sequence, which differs from GRCh37 in that *PMS2CL* sequence (chr7:6,776,750–6,791,250, highly homologous with PMS2 exon 11–15) was replaced with a string of nucleotide T to enhance alignment of the long-range PCR fragment sequence to *PMS2 *reference sequence.

In addition to SNVs and small Indels, CNVs have been implicated as the cause of inherited cancer syndromes [37]. However, accurate CNV detection in targeted NGS data remains challenging. Bias and noise in NGS coverage data, derived from various sources during library preparation, capture, and sequencing, distort the association between copy numbers and read coverages [38, 39]. Despite the excellent sensitivity and specificity of our CNV flagging algorithm, we observed that the algorithm needed to be supplemented by aCGH to achieve 100% specificity. By using the aCGH supplemented NGS algorithm on 10,068 clinical cases, we detected 57 CNV variants, of which 17 cases were single exon CNV variants (Supplemental [Supplementary-material supplementary-material-1]). We extended the CNV flagging algorithm specificity analysis on the 10,068 data set but limited the analysis to only those genes ordered for testing for each specimen. Specificity ranged from 95.5% (34-gene test, *n*=1, 923) to 100% (13 gene test, *n*=122), using the NGS CNV flagging algorithm alone (Supplemental [Supplementary-material supplementary-material-1]). Soukupova et al reported that coverage uniformity is an important factor for reliable CNV detection [40]. During assay development we performed multiple rounds of target capture RNA bait design and testing to enhance low-covered region and observed improved coverage uniformity. Further optimization of NGS library preparation procedures, such as using normalized high-quality DNA for library preparation, may improve coverage uniformity and, therefore, CNV specificity. In addition, employing recently described denoising methods based on a signal processing technique may enhance the detection accuracy of our CNV algorithm [38]. Another factor that may confound accurate CNV analysis is a processed pseudogene if its existence is unknown. The unique feature of processed pseudogenes, lacking promoter and intronic sequences, may cause false-positive exonic duplication calls. The presence of an *SMAD4* processed pseudogene was first reported in 2015 in a subset of individuals, with a frequency of 0.26% (12/4,672 clinical cases) [41]. A similar frequency (0.25%, 58/23,032 clinical cases, data not shown) was observed from our clinical results. Upon visual inspection using an Integrative Genomics Viewer (IGV), the discontinuous coverage depth pattern on exon/intron boundaries, unique to processed pseudogenes, was readily identified and aCGH confirmation gave a concordant result. In addition, no erroneous splice site variant call was made, and visual inspection with IGV confirmed retention of >98% reference sequence at each intronic position within our ROI.

Multigene panel testing can be utilized to identify individuals who are at an increased risk of hereditary cancer. Identification of an inherited risk factor allows patients options decreasing that risk, including increased surveillance, surgery, and/or chemoprevention [18, 24–26]. Targeted testing for at-risk family members can subsequently be performed. If positive, the family member can take steps to prevent cancer or aid in its early detection. Negative results can reassure the family member and prevent unnecessary surveillance or other preventive measures. For women with pathogenic/likely pathogenic variants in *BRCA1/2*, some risk-reducing strategies, such as prophylactic surgeries, have demonstrated reduced cancer risk and mortality in some studies [42–44], but not others [45]. Risk-reducing surgery for individuals with *BRCA1/2* variants and breast cancer has not yet demonstrated an overall survival benefit. Genetic information can also be used to select the patients most appropriate for targeted therapies. For example, women whose ovarian or breast cancer is associated with pathogenic variants in *BRCA1/2* may be treated with poly (ADP-ribose) polymerase (PARP) inhibitors that have been approved for the management of advanced *BRCA1/2* mutation-associated ovarian cancer [46–49].

Historically, testing for germline pathogenic or likely pathogenic variants has been performed sequentially through single-gene or single-syndrome testing. However, a multigene panel approach has a number of advantages over the traditional sequential approach. Previous studies have concluded that multigene panel testing compared with a single-gene testing can cost-effectively improve the identification of at-risk individuals for early health interventions and the outcome of cancer treatment [3, 50].

Recent studies have highlighted that panel testing is able to uncover clinically actionable variants unrelated to the syndrome that the clinician initially suspected [51, 52]. Ricker et al. showed that 7.4% (35/475) of patients with deleterious mutations detected with a multigene panel would have had negative results with a gene-by-gene testing approach [21]. Other studies using multigene panels found that over 4% of women at risk of hereditary breast cancer had mutations in genes other than *BRCA1/2,* including *PALB2, CHEK2,* and *ATM* [53, 54]. In our cohort of 49 individuals who had at least one clinically actionable finding, 46 had provided enough clinical criteria to evaluate eligibility for genetic testing based on the National Comprehensive Cancer Network (NCCN) guidelines in effect at the time of testing: 41 met *BRCA1/2*, 2 met both *BRCA1/2* and Lynch criteria, and 3 did not meet either criteria for testing, based on the information provided. Of the individuals that met NCCN criteria for *BRCA1/2 *testing, 22 (54%) had clinically actionable variants identified in genes other than *BRCA1/2* (Supplemental [Supplementary-material supplementary-material-1]). Variants were identified in genes causative of hereditary breast cancer, such as *ATM*, *BARD1*, *CDH1*, *CHEK2,* and *PALB2*. Because these genes are not associated with a single-gene syndrome, they are not typically interrogated during sequential evaluation. Currently, there are medical management guidelines for these genes, except for *BARD1 *[25]. However, variants were also identified in genes with no consensus link to hereditary breast cancer, such as *MUTYH*, *PMS2*, *RAD51C*, and *RET*. The individual found to have the pathogenic *RET* variant had no reported personal or family history indicative of any of the associated conditions. Interestingly, 8 *MUTYH* carriers were identified because they were sent for a breast/ovarian indication. In summary, pathogenic and likely pathogenic variants were identified in 8-genes that are not part of single syndrome testing for *BRCA*-related breast and ovarian cancer syndrome or Lynch syndrome.

Outside of identifying actionable variants in genes incidental to the initially suspected syndrome, previous studies have also established that the rate of families carrying more than one actionable variant is higher than what was initially thought, when guidelines recommended cascade testing for only the known familial pathogenic/likely pathogenic variants [55–57]. In our cohort of 49 individuals with actional findings, 5 had verified familial variants. If testing had been restricted to the known familial variants in these cases, the testing strategy would have failed to identify an actionable variant in one of these families, specifically a *CHEK2* c.1100delC in a family with a known *PMS2 *variant. Additionally, of the 500 individuals tested, 4 were identified as having two pathogenic variants (0.8% of the overall cohort, 8% of those who had any actionable finding). Three individuals had a pathogenic variant in both *BRCA1* and *BRCA2*, one of whom inherited both these mutations from a family member. One individual had a pathogenic variant in *BRCA2 *and in *MUTYH. *For this individual, the pathogenic variant in *MUTYH* would have been missed if testing had been restricted to a single gene/syndrome instead of pursuing panel testing. Currently, individuals with one pathogenic/likely pathogenic variant in *MUTYH* can consider early and increased surveillance for colon cancer if they have a first degree relative with colon cancer [18]. Moreover, the discovery of the single *MUTYH* variant also may have a significant impact on this individual's family members, who may need to consider increased surveillance, based on the presence of one or more clinically significant *MUTYH* variants. Individuals with biallelic pathogenic/likely pathogenic variants in *MUTYH* are associated with a lifetime risk of colon cancer of up to 80% [58, 59].

Detection of variants is more common in multigene testing due to the multiplicity of genes tested. Slavin et al summarized the results from 348 commercial multigene panel tests ordered by providers in more than 250 practice settings across the United States. A total of 348 commercial multigene panel tests ordered by providers in 2014 were reviewed and discussed during a weekly case conference. The proportion of clinically actionable results was reported as 17% for panels that included genes of both high and moderate penetrance, and 6.2% for panels that included only genes of high penetrance. When considering all variants identified in this group of patients, the proportion of VUSs was 42%. For 39% of patients, their test results were uninformative. The authors defined uninformative as an individual whose panel results revealed only benign variants and/or likely benign variants, or no variants of any classification detected. In their conclusions, the authors emphasized the importance of pre-test cancer risk assessment, due to the expected higher percentages of positive, nonclinically actionable variants, ambiguous results, and unexpected results with multigene panel testing [60].

One of the ways to reduce the number of individuals with ambiguous test results is to improve open access to variant information identified by laboratories performing genetic tests. Professional societies, including the American College of Medical Genetics and Genomics, advocate for sharing of laboratory and clinical data from individuals who have undergone genomic testing [61, 62]. Data-sharing efforts have been initiated by various organizations, including select commercial laboratories, with the goal of maintaining the privacy of patients and providers [63–65]. We are in support of open access of clinical variants identified through hereditary cancer tests, including the evidence used to determine variant classification. ClinVar (http://www.ncbi.nlm.nih.gov/clinvar/) provides a freely available archive of reports of relationships among medically important variants and phenotypes [34]. Variants identified through clinical testing at our laboratory are routinely submitted to ClinVar.

## 5. Conclusions

The 34-gene cancer predisposition panel demonstrated satisfactory performance for use in a clinical laboratory, with high sensitivity and specificity for SNVs, small indels, and exon-level CNVs. The panel can provide clinically significant information for cancer risk assessment. Multigene panel testing utilizing NGS may increase detection of pathogenic mutations compared to single-gene testing.

## Figures and Tables

**Table 1:** Genes included in the 34-gene inherited cancer predisposition panel.

Gene name	Coding reference	Genomic reference
*APC*	NM_000038.5	NG_008481.4
*ATM*	NM_000051.3	NG_009830.1
*BARD1*	NM_000465.3	NG_012047.3
*BMPR1A*	NM_004329.2	NG_009362.1
*BRCA1*	NM_007294.3	NG_005905.2
*BRCA2*	NM_000059.3	NG_012772.3
*BRIP1*	NM_032043.2	NG_007409.2
*CDH1*	NM_004360.3	NG_008021.1
*CDK4*	NM_000075.3	NG_007484.2
*CDKN2A*	NM 000077.4	NG_007485.1
	NM_058195.3	
*CHEK2*	NM_007194.3	NG_008150.2
*EPCAM*	NM_002354.2	NG_012352.2
*MEN1*	NM_130799.2	NG_008929.1
*MLH1*	NM_000249.3	NG_007109.2
*MSH2*	NM_000251.2	NG_007110.2
*MSH6*	NM_000179.2	NG_007111.1
*MUTYH*	NM_001128425.1	NG_008189.1
*NBN*	NM_002485.4	NG_008860.1
*NF1*	NM_000267.3	NG_009018.1
*PALB2*	NM_024675.3	NG_007406.1
*PMS2*	NM_000535.5	NG_008466.1
*POLD1*	NM_002691.3	NG_033800.1
*POLE*	NM_006231.3	NG_033840.1
*PTEN*	NM_000314.4	NG_007466.2
*RAD51C*	NM_058216.2	NG_023199.1
*RAD51D*	NM_002878.3	NG_031858.1
*RET*	NM_020975.4	NG_007489.1
*SDHB*	NM_003000.2	NG_012340.1
*SDHC*	NM_003001.3	NG_012767.1
*SDHD*	NM_003002.3	NG_012337.3
*SMAD4*	NM_005359.5	NG_013013.2
*STK11*	NM_000455.4	NG_007460.2
*TP53*	NM_000546.5	NG_017013.2
*VHL*	NM_000551.3	NG_008212.3

**Table 2:** SNV and Indel variants used for accuracy study.

Gene	Variant	No. specimen tested
*APC*	c.1458T > C (p.Tyr486Tyr)	2
*APC*	c.1635G > A (p.Ala545Ala)	3
*APC*	c.1958 + 8T > C (p.?)	1
*APC*	c.2547_2550del (p.Asp849Glufs^∗^11)	1
*APC*	c.3468_3470del (p.Glu1157del)	1
*APC*	c.4398del (p.Pro1467Leufs^∗^6)	1
*APC*	c.4479G > A (p.Thr1493Thr)	3
*APC*	c.5034G > A (p.Gly1678Gly)	3
*APC*	c.5268T > G (p.Ser1756Ser)	3
*APC*	c.5465T > A (p.Val1822Asp)	4
*APC*	c.5880G > A (p.Pro1960Pro)	3
*APC*	c.6281del (p.Pro2094Leufs^∗^18)	1
*BRCA1*	c.1175_1214del40 (p.Leu392Glnfs)	1
*MLH1*	c.293G > A (p.Gly98Asp)	1
*MLH1*	c.655A > G (p.Ile219Val)	1
*MSH2*	c.211 + 9C > G (p.?)	1
*MSH2*	c.1847C > G (p.Pro616Arg)	1
*MSH2*	c.2647del (p.Ile883Leufs^∗^9)	1
*MSH6*	c.116G > A (p.Gly39Glu)	1
*MSH6*	c.2633T > C (p.Val878Ala)	1
*PMS2*	c.2466T > C (p.Leu822Leu)	1
*PMS2*	c.2007−4G > A (p.?)	3
*PMS2*	c.1621A > G (p.Lys541Glu)	4
*PMS2*	c.1408C > T (p.Pro470Ser)	3
*PMS2*	c.780C > G (p.Ser260Ser)	4
*RET*	c.1826G > A (p.Cys609Tyr)	1
*RET*	c.1900T > C (p.Cys634Arg)	1
*RET*	c.2307G > T (p.Leu769Leu)	5
*RET*	c.2372A > T (p.Tyr791Phe)	1

**Table 3:** CNV positive specimens included in validation study.

Specimen name	Gene	CNV variant	No. replication
S1	*CDH1*	Exon 4–9 deletion	3
S2	*BRCA2*	Whole gene duplication	3
S3	*BRCA1*	Whole gene deletion	3
S4	*TP53*	Whole gene deletion	3
S5	*BRCA1*	Exon 16–17 deletion	3
S6	*BRCA1*	Exon 1–2 duplication	3
S7	*BRCA1*	Exon 9–12 deletion	3
S8	*BRCA1*	Exon 21–23 deletion	5
S9	*BRCA1*	Exon 13 duplication	3
S10	*BRCA1*	exon 9–12 deletion	5
S11	*MLH1*	Exon 16–19 deletion	1
S12	*PMS2*	Whole gene deletion^∗^	1
S13	*MLH1*	Exon 16–19 deletion	1
S14	*PMS2*	Exon 8 deletion	1
S15	*CDH1*	Whole gene duplication	1
S16	*BRCA1*	Exon 1–22 duplication	1
S17	*PALB2*	Exon 9–10 deletion	1
S18	*BRCA1*	Exon 22 deletion	1

^∗^S12 *PMS2* exon 11–15 CNV status was not tested by NGS.

**Table 4:** Pathogenic/likely pathogenic variants detected in the 500 consecutive specimens.

Gene	No. detected	%	Variant	Type	Classification	No. detected
*BRCA1*	12	23	c.68_69del (p.Glu23Valfs^∗^17)	DEL	P	1
			c.211A > G (p.Arg71Gly)	SNV	P	1
			c.406dupA (p.Arg136Lysfs^∗^6)	INS	P	1
			c.427G > T (p.Glu143^∗^)	SNV	P	1
			c.1960A > T (p.Lys654^∗^)	SNV	P	1
			c.3193dup (p.Asp1065Glyfs^∗^2)	INS	P	1
			c.4327C > T (p.Arg1443^∗^)	SNV	P	2
			c.5266dup (p.Gln1756Profs^∗^74)	INS	P	1
			c.5353C > T (p.Gln1785^∗^)	SNV	P	1
			c.3598C > T (p.Gln1200^∗^)	SNV	P	1
			Exon 13 Duplication	CNV	P	1

*BRCA2*	12	23	c.755_758del (p.Asp252Valfs^∗^24)	DEL	P	1
			c.1265del (p.Asn422Ilefs^∗^8)	DEL	P	1
			c.3922G > T (p.Glu1308^∗^)	SNV	P	1
			c.4284dup (p.Gln1429Serfs^∗^9)	INS	P	1
			c.4631dup (p.Asn1544Lysfs^∗^4)	INS	P	1
			c.5290_5291del (p.Ser1764Lysfs^∗^3)	DEL	P	1
			c.5385dup (p.Asp1796Argfs^∗^11)	INS	P	1
			c.5681dup (p.Tyr1894^∗^)	INS	P	1
			c.5682C > G (p.Tyr1894^∗^)	SNV	P	1
			c.5946del (p.Ser1982Argfs^∗^22)	DEL	P	2
			c.6373dup (p.Thr2125Asnfs^∗^4)	INS	P	1

*MUTYH*	9	17	c.536A > G (p.Tyr179Cys)	SNV	P	1
			c.1187G > A (p.Gly396Asp)	SNV	P	6
			c.1214C > T (p.Pro405Leu)	SNV	P	1
			c.1477G > T (p.Val493Phe)	SNV	P	1

*CHEK2*	8	15	c.444+1G > A	SNV	P	1
			c.793–1G > A	SNV	P	1
			c.1100delC(p.Thr367Metfs^∗^15)	DEL	P	5
			c.1169A > C (p.Tyr390Ser)	SNV	LP	1

*ATM*	2	4	c.7638_7646del (p.Arg2547_Ser2549del)	DEL	P	1
			c.8395_8404del (p.Phe2799Lysfs^∗^4)	DEL	P	1

*PALB2*	2	4	c.2642_2645dup (p.Cys882Trpfs^∗^3)	INS	P	1
			c.3256C > T (p.Arg1086^∗^)	SNV	P	1

*PMS2*	2	4	c.809C > G (p.Ser270^∗^)	SNV	P	1
			Exon 7–8 deletion	CNV	P	1

*CDH1*	1	2	c.1565+1G > C	SNV	P	1

*BARD1*	1	2	c.1690C > T (p.Gln564^∗^)	SNV	P	1

*CDKN2A*	1	2	c.301G > T (p.Gly101Trp) (p16)	SNV	P	1

*MLH1*	1	2	c.793C > T (p.Arg265Cys)	SNV	P	1

*RAD51C*	1	2	c.773G > A (p.Arg258His)	SNV	LP	1

*RET*	1	2	c.2410G > A (p.Val804Met)	SNV	P	1

Total	53	100				53

P, Pathogenic; LP, likely pathogenic.

## Data Availability

All relevant data are within the paper and its Supplementary Materials files.

## Supplementary Materials

Supplementary Materials Supplemental Table S1. Long-range PCR primers used in this study. Supplemental Table S2. CNV positive cases detected by aCGH supplemented NGS algorithm from 10,068 clinical cases. Supplemental Table S3. NGS CNV flagging algorithm performance evaluation on a larger data set (*n* = 10,068). Supplemental Table S4. Genetic testing eligibility based on the National Comprehensive Cancer Network (NCCN) guidelines and Pathogenic/likely pathogenic variants identified in the first 500 clinical specimens.
